# Biochemical Characterization, Thermal Stability, and Partial Sequence of a Novel Exo-Polygalacturonase from the Thermophilic Fungus* Rhizomucor pusillus* A13.36 Obtained by Submerged Cultivation

**DOI:** 10.1155/2016/8653583

**Published:** 2016-11-29

**Authors:** Lucas Vinícius Trindade, Carla Desagiacomo, Maria de Lourdes Teixeira de Moraes Polizeli, André Ricardo de Lima Damasio, Aline Margarete Furuyama Lima, Eleni Gomes, Gustavo Orlando Bonilla-Rodriguez

**Affiliations:** ^1^Instituto de Biociências, Letras e Ciências Exatas, Universidade Estadual Paulista (UNESP), São José do Rio Preto, SP, Brazil; ^2^Universidade de São Paulo (USP), FFCLRP, Ribeirão Preto, SP, Brazil; ^3^Universidade Estadual de Campinas (UNICAMP), Campinas, SP, Brazil

## Abstract

This work reports the production of an exo-polygalacturonase (exo-PG) by* Rhizomucor pusillus* A13.36 in submerged cultivation (SmC) in a shaker at 45°C for 96 h. A single pectinase was found and purified in order to analyze its thermal stability, by salt precipitation and hydrophobic interaction chromatography. The pectinase has an estimated Mw of approximately 43.5–47 kDa and optimum pH of 4.0 but is stable in pH ranging from 3.5 to 9.5 and has an optimum temperature of 61°C. It presents thermal stability between 30 and 60°C, has 70% activation in the presence of Ca^2+^, and was tested using citrus pectin with a degree of methyl esterification (DE) of 26%. *E*_*a*(*d*)_ for irreversible denaturation was 125.5 kJ/mol with positive variations of entropy and enthalpy for that and Δ*G*_(*d*)_ values were around 50 kJ/mol. The hydrolysis of polygalacturonate was analyzed by capillary electrophoresis which displayed a pattern of sequential hydrolysis (exo). The partial identification of the primary sequence was done by MS MALDI-TOF and a comparison with data banks showed the highest identity of the sequenced fragments of exo-PG from* R. pusillus* with an exo-pectinase from* Aspergillus fumigatus*. Pectin hydrolysis showed a sigmoidal curve for the Michaelis-Menten plot.

## 1. Introduction

Pectinases are enzymes involved in the degradation of pectic substances, which are complex polysaccharides present as the major components in the middle lamella and primary cell walls in plants and have high molecular weight and are negatively charged and acidic. They are present in the form of calcium pectate and magnesium pectate [1]. Pectin has three polysaccharide structures with the first one being homogalacturonan (HG) which represents the backbone of galacturonic acid residues and is linked by *α* (1 → 4) linkages [2, 3]. The second motif is rhamnogalacturonan I (RG I), which represents a branched region that includes several neutral sugars (arabinose, galactose, and mannose) as side chains of *α*-1 and two-linked residues of L-rhamnopyranose. The third structure is rhamnogalacturonan II (RG II), a branched and common domain containing an HG backbone [4]. The HG carboxyl groups can be methylated and/or acetylated in varying degrees. By definition, pectin has a minimum of 75% methylation (pectinic acid has less than that value) and it is absent for pectic acid and polygalacturonic acid [5].

Due to the high structural diversity of pectin, various types of pectinases have been described as being produced by the same organism with different mechanisms of catalysis [6]. In summary, known pectinases are classified into three groups, pectinesterases, depolymerizing pectinases, and protopectinases [7], which represent around 25% of the commercial sales of food enzymes, obtained mostly from fungi [1], although they are also produced by bacteria, insects, nematodes, protozoans, and plants [5].

Pectinases have diverse applications in industry, especially in the fruit juice extraction, in which these enzymes, by means of the middle lamella pectin degradation, act by decreasing the viscosity and turbidity of the beverage, which facilitates subsequent processes like juice filtration [8, 9]. The incorporation of pectinolytic enzymes also preserves the nutritional value, original color, and flavor [10]. In wine industry, pectinases and other enzymes such as cellulases must be added to the grape in order to promote the degradation of cell walls and facilitate the extraction of pigments such as anthocyanins and tannins as well as terpenes, which improves the color and the aromatic qualities of the product [11]. Pectinases can also be used for the extraction of vegetable oils in the absence of hexane as well as in the functional food industry where they are used in the preparation of pectic fiber, an active prebiotic for the intestinal microbiota [12]. On the other hand, textile industry uses pectinases for the removal of pectin that covers the cellulose fibers [7, 13, 14]. Looking from the perspective of plant pathogens, pectinases are among the first secreted cell wall degrading enzymes (CWDEs) [15].

The present investigation was carried out on the production and characterization of a new exo-polygalacturonase (exo-PG) obtained from the thermophilic fungus* Rhizomucor pusillus*. These kinds of enzymes are believed to act at the final stages of pectin hydrolysis [16], performing the following reaction in a sequential fashion: ((1 → 4)-alpha-D-galacturonide)_(*n*)_ + H_2_O *↔* ((1 → 4)-alpha-D-galacturonide)_(*n*−1)_ + D-galacturonate. Besides its biochemical characterization, the enzyme was purified in order to analyze its thermal stability, a characteristic that frequently determines whether an enzyme can be incorporated in an industrial process.

## 2. Materials and Methods

### 2.1. Microorganism

The thermophilic* Rhizomucor pusillus* A13.36 was obtained from the work stock cultures of the Laboratory of Applied Biochemistry and Microbiology, IBILCE-UNESP, in São José do Rio Preto, São Paulo, Brazil. The fungus was originally isolated from decaying vegetables collected in the same city and identified in the Laboratory of Microbiology DRM/CPQBA-UNICAMP in Paulínia, SP. The strain was maintained on potato dextrose agar (Oxoid).

### 2.2. Culture Medium

A pectin source was used for the culture medium. Orange bagasse was obtained from a donation from a local firm “Jet Suco” and the wheat bran was purchased from a local market. Both materials were washed with distilled water until the reducing sugars were no longer detectable, were dried at 60°C for 40 h, and were ground and sieved to select particles between 0.1 and 0.6 mm.

Submerged cultivation (SmC) was performed in 250 mL Erlenmeyer flasks containing 40 mL of sterile liquid medium composed of the following (g/L): 10 orange bagasse, 10 wheat bran, and a nutrient solution (g/L) that consisted of 1.0 (NH_4_)_2_SO_4_, 1.0 MgSO_4_·7H_2_O, 0.2 ZnSO_4_·7H_2_O, 0.11 H_3_BO_3_, 0.05 MnCl_2_·4H_2_O, 0.05 FeSO_4_·7H_2_O, 0.016 CoCl_2_·5H_2_O, 0.016 CuSO_4_·5H_2_O, 0.011 (NH_4_) 6Mo_7_O_24_·4H_2_O, and 0.5 EDTA. The medium was sterilized at 120°C for 30 minutes and inoculated with a spore suspension volume that was equivalent to 10^7^ spores/mL. The cultivation took place in an orbital shaker at 100 rpm at 45°C for 96 hours. After cultivation, the fermented material was filtered and then centrifuged at 10,000 ×g at 4°C. The supernatant was used as crude enzyme solution for characterization and purification.

### 2.3. Enzyme Assay

The pectinolytic activity was assayed in a mixture composed of 225 *μ*L of 1% citrus pectin (26% degree of esterification) solution, 0.2 M sodium acetate buffer, pH 5.0, and 25 *μ*L of enzyme solution. The reaction rate was linear for up to 4 minutes, which was the time adopted as incubation time for the assays. The usual assay was done at 60°C and the reaction was stopped by adding 250 *μ*L of 3,5-dinitrosalicylic acid (DNS) [17]. A unit of enzyme activity was defined as the amount of enzyme required to release one *μ*mol of reducing sugar (galacturonic acid) per minute [18].

### 2.4. Enzyme Purification by Salting-Out

The enzyme was purified by salting-out [19] using an aliquot of 10 mL of crude enzyme solution which was precipitated with 95% (NH_4_)_2_SO_4_ (AS) at room temperature under continuous stirring. Acetate buffer (50 mM) and 2 mM EDTA were added previously to the enzyme solution. After salt dissolution, the mixture was left for 30 min at room temperature (25°C) and subsequently centrifuged at 3,000 ×g for 40 min. Once the precipitate was removed, approximately 12 mL of the supernatant was obtained and was subsequently dialyzed before performing the protein concentration determinations and the enzyme assay. The samples were desalted overnight at 4°C against water in 3 kDa cutoff cellulose acetate dialysis tubing (Sigma-Aldrich).

### 2.5. Hydrophobic Interaction Chromatography (HIC) for Sequencing by MALDI-TOF Mass Spectrometry

Approximately 1 mL of the 95% supernatant was applied without dialysis to a 1 mL HiTrap™ Phenyl HP column GE which was attached to an Äkta Purifier FPLC UPC10 system (*GE Healthcare Life Sciences*). The initial buffer used was 50 mM sodium acetate containing 95% (NH_4_)_2_SO_4_ and 2 mM EDTA, pH 5.0. The final buffer was 50 mM sodium acetate, pH 5.0. The flow rate used was 1 mL/min, the fractions collected were of 0.5 mL, and the elution profile was monitored by absorbance at 280 nm. The fractions with pectinolytic activity had their content dialyzed to make it possible to monitor the purification process which presented a single band corresponding to the enzyme.

### 2.6. Total Protein Quantification

The protein quantification was carried out by the binding dye method [20] and the standard curve was prepared using bovine serum albumin (Sigma-Aldrich).

### 2.7. Electrophoretic Analysis: SDS-PAGE

A 1 mL sample of the AS precipitated protein was mixed with chilled 100% trichloroacetic acid (TCA), centrifuged at 14,000 ×g, and washed twice with chilled acetone using similar centrifugation. The pellet was dissolved in 40 *μ*L of bromophenol blue containing 20% glycerol.

The protein profile was analyzed by SDS-PAGE [21, 22]. The running gel was 8%, with a 5% stacking gel, using 25 mM Tris and 0.2 M glycine (pH 8.3) buffer containing 10% (w/v) SDS.

### 2.8. Electrophoretic Analysis: Zymogram

The zymogram for exo-PG followed the protocol proposed by Polizeli et al. [23].

### 2.9. Characterization of the Pure Pectinase: Effect of pH and Temperature on Pectinolytic Activity

PG activity was assayed in a pH range from 3.5 to 9.5 using 0.1 M sodium citrate (pH 2.0 to 3.0), sodium acetate (pH 3.5 to 5.5), MES (pH 6.0 and 6.5), HEPES (pH 7.0 to 7.5), glycine (pH 8.0 to 9.5), and CAPS (pH 10.0 to 11.0) containing 1% citrus pectin (degree of esterification 26%). When necessary, the pH of the buffers was adjusted to correct for the displacement effect of p*K*a depending on the temperature following tabulated values of Δp*K*a/°C [24].

The pectinolytic activity was tested as a function of temperature (30 to 80°C) at the optimum pH using 1% citrus pectin (degree of esterification 26%).

The effect of the pH on stability was evaluated by mixing a 1 : 1 ratio of the enzymatic solution of 0.1 M in the same buffers described above and incubating this mixture in the absence of substrate for 24 h at 25°C. Residual activity was measured at optimum pH and temperature. To calculate the residual activity, a sample was used as a 100% control and was prepared by mixing (1 : 1) the enzyme solution in water and incubating the mixture at room temperature for 24 h in the absence of the substrate.

Irreversible thermal denaturation and thermal stability of exo-PG were performed in the absence of the substrate after incubation for one hour in temperatures ranging from 30 to 80°C and keeping the samples overnight in ice before performing the enzyme assays and quantifying the residual pectinolytic activity. All the calculations were performed following the guidelines provided by the available literature [25–27].

### 2.10. Cation Effect on the Activity of the Partially Purified Pectinase

Before performing this experiment, gel filtration on Sephadex G-50 was done to remove a large part of the contaminants of lower molecular mass in order to avoid interference of salts already present in the extract. The partially purified enzyme was used to test the effects of various cations on the enzyme activity at a final concentration of 10 mM individually to the enzyme assay (NaCl, MgCl_2_, AlCl_3_, KCl, FeCl_3_, CaCl_2_, BaCl_2_, and MnCl_2_).

### 2.11. Functional Characterization of the Pure Pectinase

The identification of enzymatic hydrolysis products was carried out by capillary electrophoresis and derivatization with 8-aminopyrene-1,3,6-trisulfonic acid trisodium salt by reductive amination. The enzymatic reactions took place for up to 12 h using polygalacturonic acid as a substrate. The capillary electrophoresis of products generated was performed on a P/ACE MQD (Beckman Coulter) with laser-induced fluorescence detection using a fused silica capillary (50 *μ*m in diameter) as a separation column. The emission was collected through a filter that was in the 520 nm range.

### 2.12. Kinetic Studies

The experiments for analyzing the variation of the initial rate of hydrolysis *V*_*o*_ as a function of substrate concentration and estimation of the apparent kinetic parameters (*V*_max_ and *K*_*m*_) of the pectinase were performed using with the substrate citrus pectin DE 26% (Sigma-Aldrich), which was dissolved in a sodium acetate buffer 0.2 M with pH of 4.0 at concentrations ranging from 0.4 to 8 mg/mL.

### 2.13. Estimation of the Molecular Mass of the Pectinase under Native and Denaturing Conditions

The estimated molecular mass under denaturing conditions was assessed by SDS-PAGE. Comparison was done between globular proteins standards in a graph where the logarithm of the molecular mass versus the relative migration in the gel was plotted [28]. Proteins with Mw lower than 29 kDa were excluded from the plot. The Wide Range kit from Sigma-Aldrich was used.

The Mw estimation for the native enzyme was done by gel filtration chromatography in a GE XK-16/100 column filled with Sephadex G-75 attached to an Äkta Purifier FPLC UPC10 system (*GE Healthcare Life Sciences*). The molecular mass was estimated by plotting a graph of the logarithm of the molecular mass of globular protein standards of known molecular weight which were carbonic anhydrase (29 kDa), myoglobin (17.8 kDa), and serum albumin (66 kDa) versus the partition coefficient (*K*_av_). Blue Dextran was used to determine the exclusion volume *V*_*o*_. The total volume was related to the gel geometric volume and *V*_*t*_ = *r* ^2^ × *π* × *l* equation was used in which *r* is the radius and *l* is the column length, respectively. The distribution coefficients of the standards were calculated by the following equation [29]: *K*_av_ = (*V*_*e*_ − *V*_*o*_)/(*V*_*t*_ − *V*_*o*_), with the elution volumes expressed in mL, where *V*_*e*_ represents the elution volume of the absorbance peak at 280 nm of each protein standard and exo-PG.

### 2.14. Partial Sequencing of the Purified Pectinase by Mass Spectrometry

Samples obtained from HIC were prepared and applied to 10% SDS-PAGE where the gel was stained with Coomassie Brilliant Blue G-250. The band was cut and sent to the National Laboratory of Science and Technology of Bioethanol (CTBE) at Campinas, SP (Brazil), for mass spectrometry analysis. At CTBE, the samples were reduced with DTT and alkylated with blocked iodoacetamide before cleavage with trypsin.

Using the data of MS, MS/MS, and Mascot, a database site for the interpretation of mass spectra, the samples were identified with mass tolerance of ±0.02 Da. The parameters of peptides and mass tolerance of the fragment were ±0.5 Da. The instrument used was a MALDI-TOF/TOF.

## 3. Results and Discussion

### 3.1. Pectinase Production

**Figure 1:**
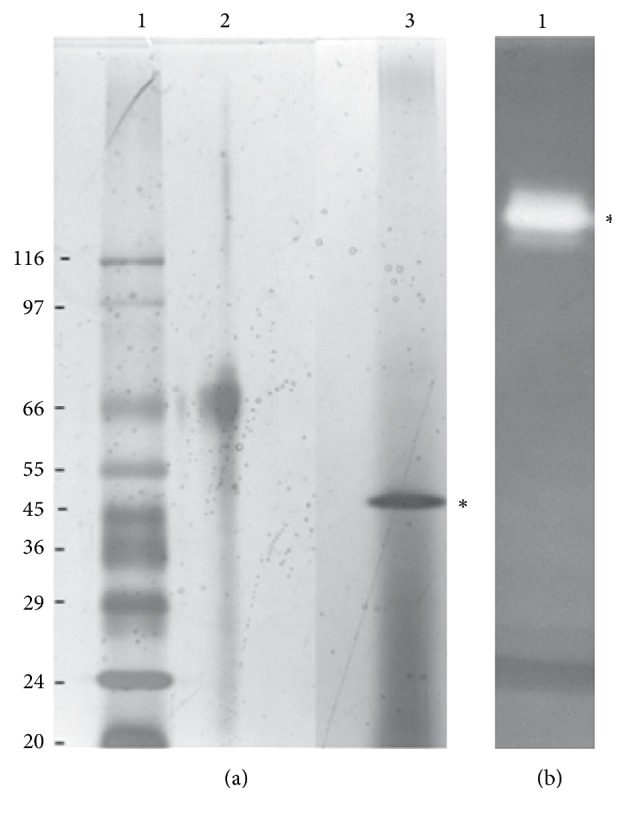
(a) SDS-PAGE gel (8% running gel and 5% stacking gel). Lane 1: profile of molecular weight markers (values in kDa). Lane 2: BSA, bovine serum albumin. Lane 3: profile of the crude extract, with the asterisk marking the spot corresponding to the exo-PG. (b) Nondenaturing gel of the crude extract (12% running native gel and 5% stacking gel). Lane 1: the band corresponding to exo-PG.

The maximum production of the enzyme occurred at 96 hours of cultivation (results not shown). A single band was evident (Figure 1) by zymography and the different position of the band was because the concentration and shape of the polyacrylamide gels were different for the SDS-PAGE and the zymogram.

**Figure 2:**
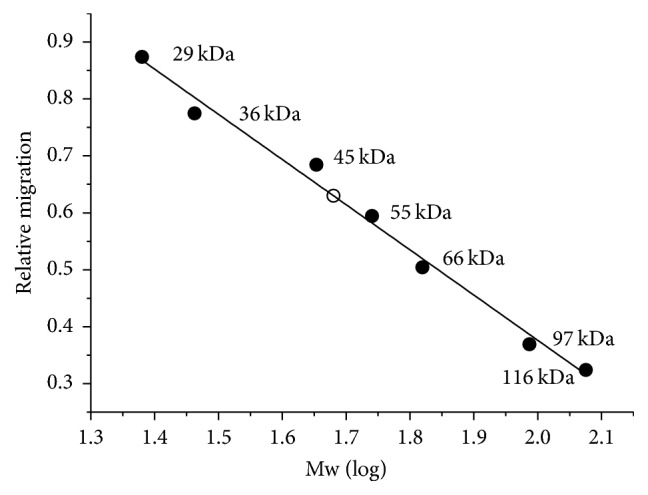
Estimation of the molecular mass of the exo-PG (empty circle) from* R. pusillus* by SDS-PAGE by comparison with the relative migration of globular proteins used as markers (filled circles).

Under denaturing conditions (SDS-PAGE), the migration of the pectinase was compared to the corresponding movement of standard globular proteins (Figure 2). The molecular mass was estimated at approximately 47 kDa using linear regression. The molecular mass of the native enzyme was also calculated from gel filtration experiments using Sephadex G-75 as being approximately 43.5 kDa by means of a similar plot using known globular proteins as calibration markers as described (results not shown).

### 3.2. Cation Effect on the Activity of the Partially Purified Pectinase

Sodium did not significantly influence the pectinolytic activity (Table 1), but other cations such as Mg^+2^, Fe^+3^, Ba^+2^, and Mn^+2^ displayed significant inhibition of the enzyme under study even though some of them were present in the original nutrient solution. Other elements such as Al^+3^ and especially Ca^+2^ proved to activate the enzyme. The ion Ca^+2^ increased its activity by 70%. The pectinolytic activity of* Penicillium viridicatum* in the presence of the same cation increased by 10–30% at concentrations of 2 and 5 mM but was only 7% at 10 mM which was the concentration that was tested [30]. The exo-polygalacturonase from* Aspergillus sojae* [31] was not activated by calcium but by zinc which produced an increase of only 12% of the activity.

The analysis of the purification process and the subsequent characterization of the pure enzyme were carried out with the addition of 10 mM of CaCl_2_ in the assay mixture in order to provide the enzyme cofactor and ideal conditions for catalysis.

### 3.3. Purification of the Pectinase

The enzyme was initially purified with 95% ammonium sulfate which maintained the supernatant substantially free of contaminants (results not shown). Kosmotropic salts such as those used in the precipitation sequester water molecules which enable the interactions between hydrophobic groups and the induction of protein precipitation [29]. Since the pectinase did not precipitate under those conditions, we assume that it probably has small hydrophobic patches close to the surface or that interference of linked oligosaccharides occurs.

The technique proved that the purification of the enzyme was fast and cost efficient and ensured a high yield (Table 2). The purity and yield of the enzyme after salting-out was performed were considered sufficient to continue the studies of biochemical characterization, kinetics, and thermodynamics. The second step of purification by hydrophobic interaction chromatography (HIC) was performed for MALDI-TOF since it required a higher degree of purity. The final yield was presumably low by the loss of activity after HIC.

### 3.4. Biochemical Characterization of the Pectinase

**Figure 3:**
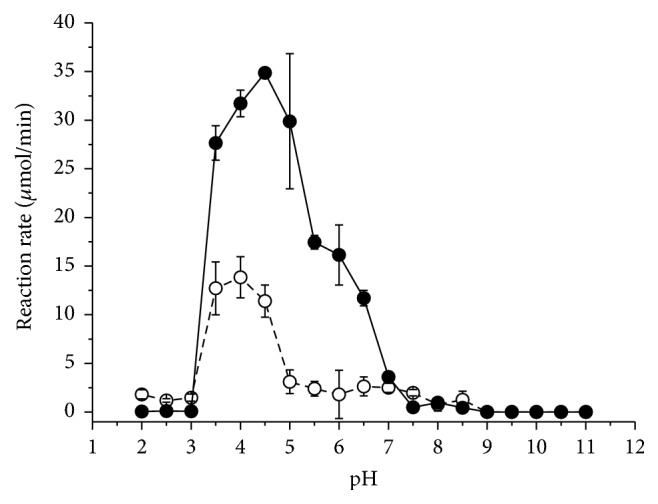
Effect of the pH on the exo-PG of the crude extract of* R. pusillus* (filled circles) and the pure enzyme (empty circles). The symbols represent the mean values and the bars represent the standard deviation.

The optimum pH of the pectinase was 4.5 for the enzyme in the crude enzyme solution and 4.0 for the pure enzyme (Figure 3) with the optimum temperature being around 60°C for both. Usually, thermozymes present optimal temperatures in the range of 60 to 80°C [32].

**Figure 4:**
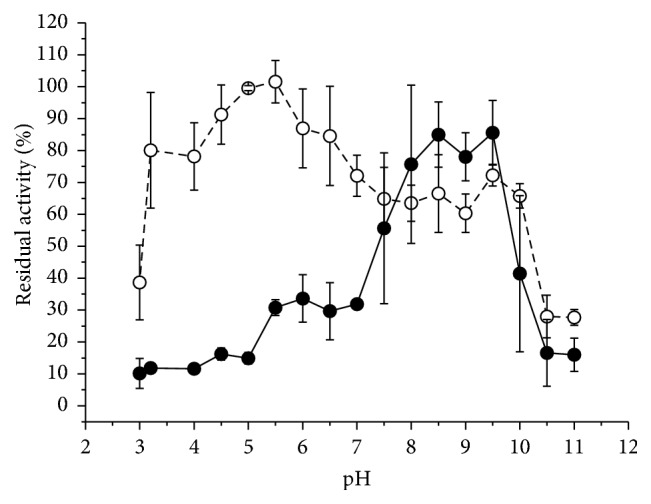
Residual activity of the exo-PG from* R. pusillus* when incubated at different pH values for 24 hours at room temperature in the absence of substrate. Filled circles: crude extract; open circles: pure enzyme. The residual activity was calculated from a control sample that was prepared by mixing (1 : 1) the enzyme solution in water and was incubated at room temperature for 24 hours in the absence of the substrate (control = 100%). The average (symbols) and standard deviation (bars) are reported in the graph.

The crude pectinase displayed stability at pH values ranging from 8 to 9.5 (Figure 4). This is remarkably different from those found for the pure pectinases after contaminants were removed which remained stable at pH ranging from 3.5 to 6.5.

**Figure 5:**
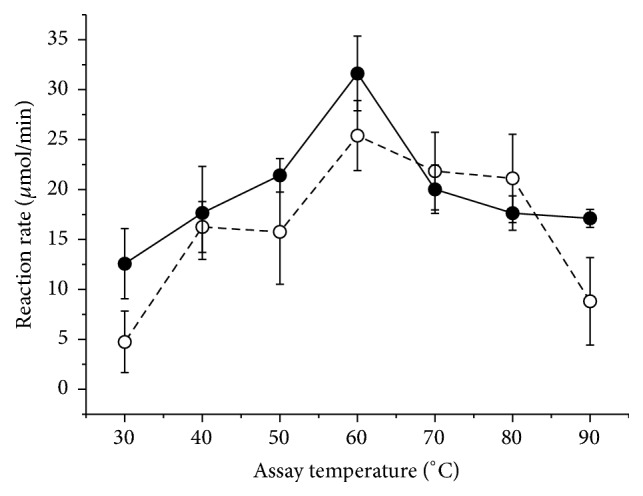
Effect of the incubation temperature on the activity of the exo-PG from* R. pusillus*. Filled circles: crude extract; open circles: pure enzyme. In the graph, the average (symbols) and standard deviation (bars) are represented.

**Figure 6:**
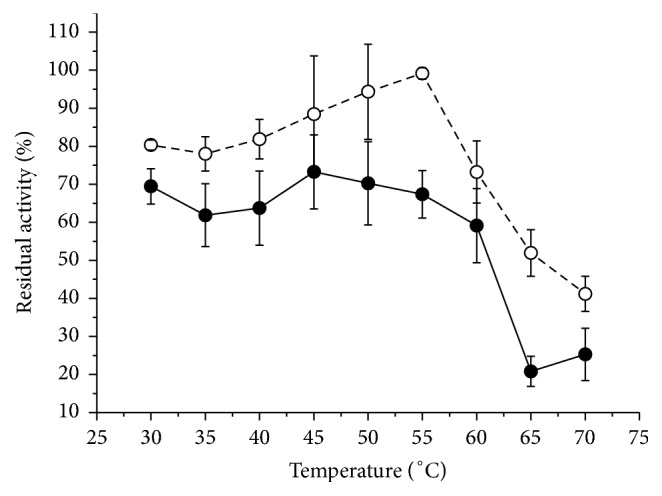
Residual activity of the exo-PG when incubated at different temperatures for 1 hour in the absence of substrate (filled circles: crude extract; open circles: pure enzyme). The residual activity was calculated from a control sample which was not incubated at different temperatures (control = 100%). The average (symbols) and standard deviation (bars) are reported in the graph.

Figure 5 shows that the optimum temperature for the purified and nonpurified enzymes is around 60°C and any value above that would denature the enzyme. The pectinase remained stable when incubated for 1 hour in the absence of substrate at 60°C and maintained its residual activity (Figure 6). High rigidity of thermophilic enzymes derives from folding stability and requires a high-temperature activity (generally greater than 40°C) to promote thermal movement and increased structural flexibility that is essential for catalysis [33].

### 3.5. Kinetic Studies

**Figure 7:**
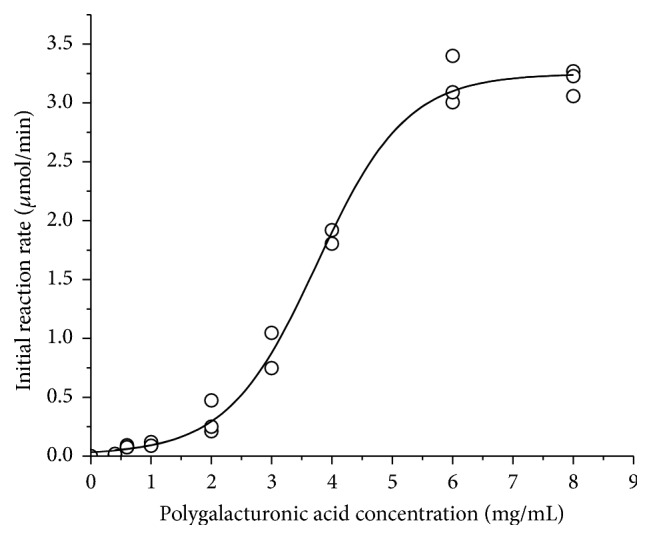
Michaelis-Menten plot for the purified exo-PG from* R. pusillus*.

Figure 7 represents the variation of the enzymatic activity at different pectin (DE 26%) concentrations. The obtained curve was clearly not hyperbolic but sigmoidal and it was not possible to find similar data in the literature to compare it. We believe that this is due to the fact that the substrate is a complex polymer and not a synthetic substrate, which would be ideal for kinetic analysis. We hypothesize that some regions of the substrate offer an initial difficulty for the enzyme and hydrolysis that occurred at lower rates but as hydrolysis proceeded the effectiveness increased as suggested by the increase of the steepness in the curve. Although sigmoidal curves for the initial rate versus substrate concentration are typical of allosteric enzymes, it does not make sense in this case, since, with pectinase being a monomeric enzyme, it would be unlikely to display cooperativity [34], although there are exceptions [35]. Therefore, the Michaelis-Menten constant (*K*_*m*_) and maximum reaction rate *V*_max_ were not estimated.

### 3.6. Thermodynamics of Irreversible Thermal Inactivation

**Figure 8:**
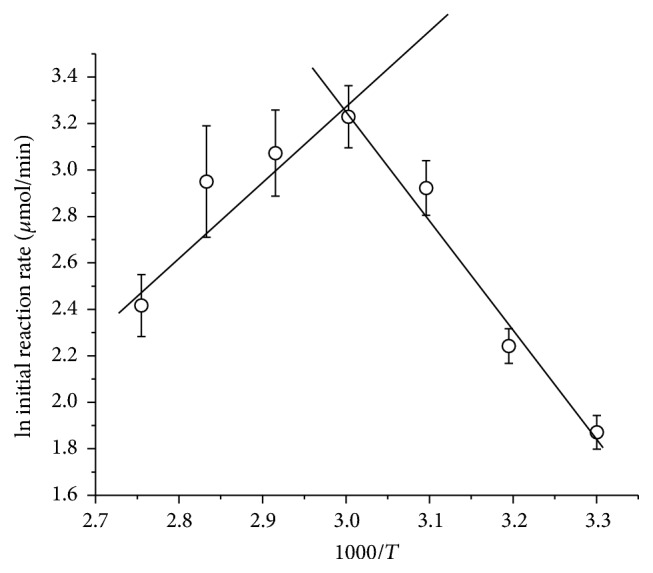
Arrhenius plot to calculate the activation energy (*Ea*) and optimum temperature of pure exo-PG from* R. pusillus*. The average (symbols) and standard deviations (bars) are reported in the graph.

The Arrhenius curve (Figure 8) allowed the estimation of the optimum temperature and the activation energy (*Ea*) of the pectinase. The temperature coefficient (*Q*_10_), which represents the increase in the reaction rate every 10 degrees Celsius of temperature rise, was also estimated at different temperatures. The parameters are listed in Table 3.

The low enzyme activation energy (15.1 kJ/mol) indicates that the pectinase must overcome a small energy barrier to hydrolyze the substrate which resulted in a lower value than those reported for two endoglucanases from* Aspergillus fumigatus* [27], and *Q*_10_ values at 50°C (1.57) are also intermediate compared to those enzymes (1.46 and 1.80) under the same conditions. The *Q*_10_ values showed a gradual decrease as the temperature arose which was a result of thermal denaturation.

**Figure 9:**
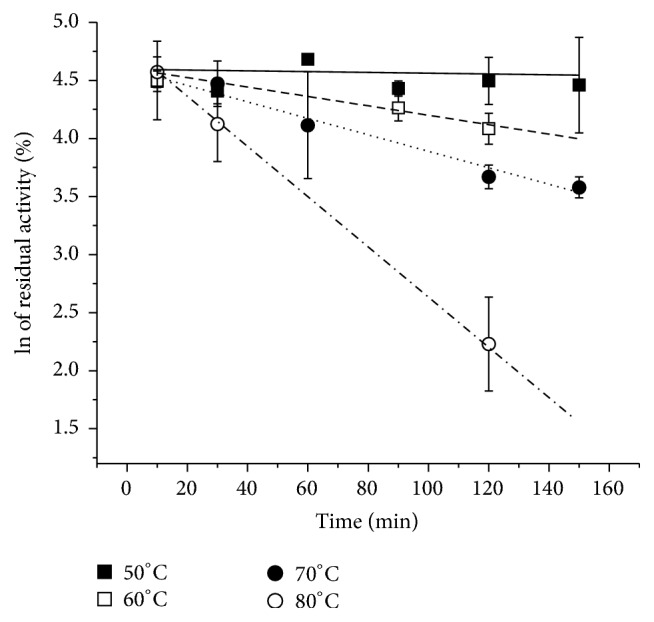
First-order plot of the effect of temperature on the activity of the pure exo-PG between 50 and 80°C. The average (symbols) and standard deviations (bars) are reported in the graph.

Figure 9 shows the effect of incubation time at different temperatures (from 50 to 80°C) in the pectinolytic activity. Other thermodynamic parameters are described in Table 4. In comparison with results available in the literature, it is observed that the half-life time at 60°C was longer than that described for the exo-PG from* Thermomucor indicae-seudaticae* [36]. The results obtained were 161.2 min and 58.3 min at 55°C for submerged and solid cultivation, respectively, which indicate high thermal stability of the exo-PG. Some pectinases from commercial preparations have reported very small half-lives such as 17 minutes at 50°C [37].

**Figure 10:**
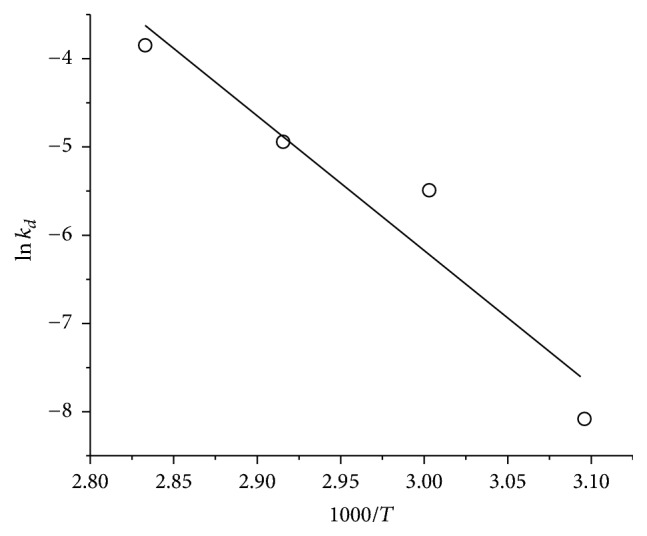
First-order Arrhenius plot for the determination of activation energy of denaturation (*E*_*a*(*d*)_) of pure exo-PG from* R. pusillus*.

The activation energy of thermal denaturation *E*_*a*(*d*)_ calculated in Figure 10 (125.5 kJ/mol) represents the energy barrier required to lead the enzyme to an irreversibly denatured state in *N*↔*D* → *I* balance between the native and denatured states which can lead to irreversible denaturation “*I*.” The reported values for the endoglucanases from* A. fumigatus* obtained by submerged cultivation and solid state were 154.7 and 114.3 kJ/mol, respectively [27], while a higher value of 286.2 kJ/mol for the exo-PG from* A. sojae* was reported [31]. Positive Δ*H*_(*d*)_ values were obtained, indicating the endothermic nature of the denaturation reaction. The Δ*H*_(*d*)_ values are intermediate with respect to those reported for endoglucanases from* A. fumigatus* (112 and 152 kJ/mol) [27], but lower than 357 kJ/mol for an endoglucanase from* A. oryzae* [38].

Entropy values refer to the degree of solvation and the compactness of the protein molecule while the increase in entropy suggests opening up of the enzyme structure [25]. In general, the variation of entropy is positive due to an increase in disorder, while low values of Δ*S*_(*d*)_ suggest the exposure of nonpolar side chains which causes the order of water molecules in the form of clathrates or cages [39]. The positive change in entropy of denaturation was above 200 J/(mol·K) which is less than the reported value for an endoglucanase from* A. fumigatus* [26] which is in the range of 400 J/(mol·K) and very different from the values obtained for a carboxymethylcellulase from* A. niger*, situated between −200 and −210 J/(mol·K) [39], and for a *β*-glucosidase from* F. solani*, with values around −170 J/(mol·K) [40].

Regarding the Gibbs free energy variation Δ*G*_(*d*)_, the values calculated in this work are around 50 kJ/mol and are lower than those reported for the pectinase from the bacteria* Erwinia carotovora* [41] which were about 85 kJ/mol. The values tend to increase as temperature rises, a phenomenon also verified for an invertase from* F. solani* [25].

### 3.7. Substrate Specificity and Hydrolysis Products by Capillary Electrophoresis

The assays using substrate with different esterification degree confirm the higher activity of the enzyme on pectin with 26% of esterification compared to 82%. The capillary electrophoresis of products of hydrolysis of polygalacturonic acid showed a pattern of action “exo,” hydrolyzing the end of the substrate chain, since it was detected as release of monogalacturonic acid (results not shown).

### 3.8. Analysis of Exo-PG by Mass Spectrometry

**Figure 11:**
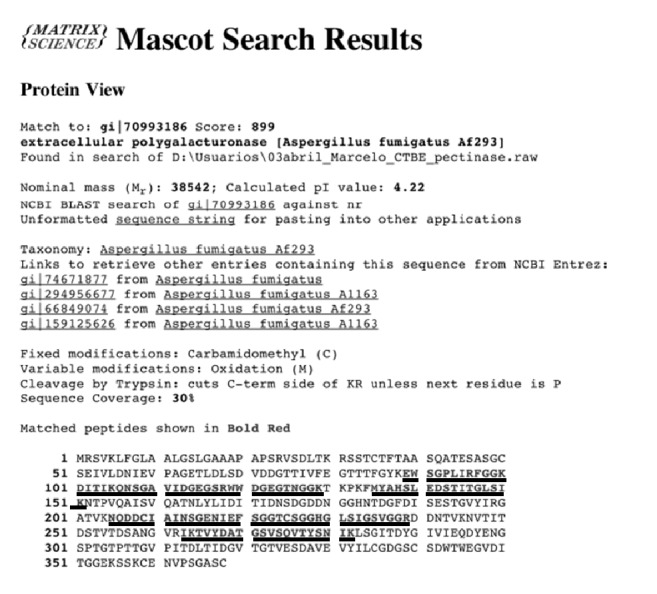
Results of sequencing of exo-polygalacturonase* R. pusillus* by mass spectrometry. The underlined residues were originally in red and correspond to those identical to the sequence of an exo-PG from* Aspergillus fumigatus*.

The analysis of the sequence of the purified PG (Figure 11) was performed by mass spectrometry using MALDI-TOF and the fragments were compared to a database, and a 30% identity with an exo-polygalacturonase from the fungus* A. fumigatus* was found. Part of the fragments from* R. pusillus* exo-PG contained presumably linked carbohydrates, not allowing performing a full comparison. Identical residues are underlined in the sequence, belonging to* A. fumigatus*. The molecular mass of* A. fumigatus* enzyme is 38.5 kDa, which is lower than the estimates made by denaturing gel filtration and electrophoresis for the exo-PG of this work, but the differences can be attributed to the glycosylation of* R. pusillus* exo-PG. Of the four conserved motifs listed by Palanivelu, three are found in the underlined sequence. These are G/QDD (206–208), G/SHG (228–230), and RIK (262–264) which are in the same positions occupied in a polygalacturonase from* A. niger* (UniProt P26213). The last one is in fact confirmed to be identical in Ile and Lysine. The two first regions cited above are catalytic [42]. Three aspartates are considered to be essential for the acid/base mechanism which was confirmed by site-directed mutagenesis [16]. The same authors, analyzing the crystal structure of the exo-polygalacturonase from* Yersinia enterocolitica*, observed that the topography of the active site prevents binding to internal residues of polygalacturonic acid.

## 4. Conclusion

In conclusion, the single pectinase of* R. pusillus* was easily purified and is a catalytically efficient enzyme for polygalacturonic acid hydrolysis. Its kinetic and thermodynamic properties suggest that the enzyme would be efficient for industrial purposes, motivating us to perform tests to evaluate its potential for specific applications.

## Figures and Tables

**Table 1:** Effect of cations (final concentration 10 mM) on the relative activity (%) of the exo-PG from *R. pusillus* partially purified. The asterisks designate significant differences between arithmetic means by Student's *t*-test (*p* < 0.05) ± standard deviation. The experiments were performed at pH 5.0 at 60°C.

Ions (10 mM)	Mean (%)		SD (%)
Control	100.0	±	0.8
NaCl	95.2	±	8.0
MgCl_2_	34.1^*∗*^	±	3.5
AlCl_3_	125.4^*∗*^	±	7.8
KCl	96.2^*∗*^	±	0.7
FeCl_3_	0.0^*∗*^		—
CaCl_2_	170.3^*∗*^	±	0.8
BaCl_2_	24.5^*∗*^	±	6.5
MnCl_2_	0.0^*∗*^		—

**Table 2:** Purification table. The yield calculation considered as the initial condition the crude extract (100%). To calculate the purification factor, the initial condition (crude extract) with a purification factor 1 was considered. The salting-out condition refers to the supernatant obtained with 95% saturation of ammonium sulphate, and HIC stands for hydrophobic interaction chromatography.

Purification step	Volume (mL)	Total activity (U)	Total protein (mg)	Yield (%)	Specific activity (U/mg)	Purification factor
Crude extract	10.0	170.0	1.10	100.0	149.0	1.0
*Salting-out*	40.8	124.2	0.50	73.5	223.4	1.4
HIC	15.0	25.2	0.15	14.9	171.0	1.2

**Table 3:** Activation energy (*Ea*), optimum temperature, and temperature coefficient (*Q*_10_) estimated based on Figure 8.

Parameter	
*E*_*a*_ (kJ/mol)	15.1 ± 3.8
*Q*_10_	1.57 (50°C); 1.53 (60°C); 1.50 (70°C); 1.46 (80°C)
Optimum temperature (°C)	61.1

**Table 4:** Kinetic and thermodynamic parameters of the irreversible thermal inactivation.

Temperature (°C)	Temperature (K)	*k*_*d*_ (min^−1^)	*T*_1/2_ (min)	Δ*H*_*d*_ (kJ/mol)	Δ*G*_*d*_ (kJ/mol)	Δ*S*_*d*_ (J/mol·K)
50	323	0.0003	2310.0	124.7	47.8	238.2
60	333	0.0041	169.0	124.6	49.3	226.1
70	343	0.0072	96.3	124.5	50.9	214.7
80	353	0.0213	32.5	124.4	52.4	203.9
